# Anxiety, Depression, and Apathy as Predictors of Cognitive Decline in Patients With Parkinson's Disease—A Three-Year Follow-Up Study

**DOI:** 10.3389/fneur.2022.792830

**Published:** 2022-02-08

**Authors:** Ketevan Toloraia, Antonia Meyer, Selina Beltrani, Peter Fuhr, Roselind Lieb, Ute Gschwandtner

**Affiliations:** ^1^Department of Neurology, University Hospital Basel, Basel, Switzerland; ^2^Division of Clinical Psychology and Epidemiology, Department of Psychology, University of Basel, Basel, Switzerland

**Keywords:** anxiety, mild cognitive impairment, Parkinson's disease, neuropsychology, neuropsychiatry

## Abstract

**Objective:**

Anxiety, depression, and apathy are the most common neuropsychiatric symptoms in Parkinson's disease (PD) patients. They impair cognitive functioning and have a profound impact on quality of life. This follow-up study aims to investigate the predictive value of anxiety, depression, and apathy on the development of Mild Cognitive Impairment (MCI) in PD patients.

**Methods:**

Twenty-nine cognitively unimpaired PD patients (mean age 68.2 SD ± 7.12 years; 13 women) participated in this study. At Baseline (BL) levels of apathy (Apathy Evaluation Scale, AES), depression (Beck Depression Inventory, BDI-II), and anxiety (Beck Anxiety Inventory, BAI), were assessed. Cognitive status was reassessed three years later according to MCI/non-MCI status. For statistics, we used binary logistic regression and receiver operating characteristic curve (ROC) analysis to examine anxiety, apathy, and depression at BL as a predictor of MCI status three years later.

**Results:**

Eight of the 29 patients developed MCI. Anxiety level at BL was found to predict MCI status at three-year follow-up (*OR* = 1.20, *CI* = 1.02–1.41, *p* = 0.02), while depression (*OR* = 1.16, *CI* = 0.93–1.47, *p* = 0.20) and apathy (*OR* = 1.06, *CI* = 0.92–1.23, *p* = 0.40) did not predict MCI status. The area under the ROC curve (AUC) of BAI for discriminating PD-non-MCI from PD-MCI was 0.79 (*CI* = 0.61–0.98). The optimal classification threshold yielded a sensitivity of 75.0 % and a specificity of 76.2 %. Neither apathy nor depression at BL discriminated between PD-non-MCI patients from PD-MCI three years later.

**Conclusions:**

This study shows an association between anxiety and the development of MCI in PD patients, although the association between apathy, depression, and MCI did not reach a significant level.

## Introduction

Non-motor symptoms (NMS) in patients with Parkinson's disease (PD), such as neuropsychiatric symptoms, sleep disturbances, autonomic dysfunctions, and sensory impairments, can occur more than a decade before the onset of motor symptoms. They progress slowly; and, may add up to a major disability. NMS are common in all stages of Parkinson's disease (1). Often they have a greater impact on the quality of life of patients and caregivers than motor symptoms (2, 3). As another NMS, ~27 to 53% of PD patients develop mild cognitive impairment (MCI), which is defined as an intermediate stage between unimpaired cognition and the state of dementia (4). Even if MCI does not yet negatively affect patients‘ daily activities, it can be objectified by standardized cognitive tests. Up to 80% of PD patients with MCI further progress to a state of Parkinson's disease dementia (PD-D) (5). Cognitive impairment is more commonly detected in the relatively late stage of this disease (2). While anxiety, apathy and depression are major neuropsychiatric symptoms of PD that emerge earlier than motor symptoms, anxiety, is one of the most common neuropsychiatric symptom with a comorbidity rate of 20 to 65% (6, 7). Additionally, anxiety may have a greater impact on the quality of life than depression and apathy (7).

The most common manifestations of anxiety in PD patients are panic attacks, social anxiety, and generalized anxiety disorder (GAD) (8). Anxiety can exacerbate motor symptoms (9), as well as increase self-perceived weakness (3). Furthermore, in addition to depression, characterized by loss of interest, difficulty concentrating and sad mood, and apathy, defined as lack of motivation, and reduced goal-directed behavior, anxiety can have a negative impact on cognitive functioning (9–11).

PD patients with MCI are more likely to experience neuropsychiatric symptoms, including anxiety (12), apathy (10), and depression (9).

The studies showed different results regarding the association between MCI and anxiety, depression, and apathy in PD patients. One study found that depression and anxiety were more common in PD patients with MCI (13), alternatively, another study reported that apathy was an important neuropsychiatric symptom, which distinguished PD-MCI from PD-NC (14).

This study aims to investigate whether future development of MCI is predicted by Baseline (BL) levels of anxiety, depression and/or apathy in a longitudinal study (2).

## Methods

### Participants

A total of 81 patients with idiopathic PD were recruited from the Basel training studies (15, 16) at the University Hospital Basel, Switzerland. Data was collected from March 2011 to May 2017. The patients completed a comprehensive neuropsychological test battery and a neuropsychiatric and neurological examination was performed. The criteria for inclusion were diagnosis of idiopathic PD according to the UK Parkinson's Disease Brain Bank Criteria (17) and written informed consent. At the time of their participation in the study, all patients were taking dopaminergic medication and were tested in their “ON” state.

Patients were excluded if they had MCI, based on the Movement Society Task Force Level II criteria according to Litvan et al. (5) insufficient knowledge of the German language, severe neurological disease other than PD.

The local ethics committee approved the studies (Ethikkommission beider Basel, Reference: 135/11 and 294/13). All participants were fully informed of the nature of the studies and gave written informed consent.

In the present follow-up study, we focused on PD patients with normal cognition (NC) at BL only. These patients were reexamined three years later to analyze their cognitive status as a function of non-MCI/MCI status. Of the 81 patients recruited, fifty-two had previously been excluded because either they had MCI at BL (*n* = 35), or their MCI status had not been assessed at BL or follow-up (*n* = 15), or they had been diagnosed with dementia three-year follow-up (*n* = 2). Accordingly, 29 cognitively unimpaired PD patients (13 female, 16 male) were included in the present study.

### Study Design

This prospective longitudinal study, which includes 29 initially cognitive unimpaired idiopathic PD patients, examined the risk factors for MCI over a three-year follow-up period. The predictor variable at BL was each patient's BAI, BDI-II, and AES BL score. The outcome variable was their MCI status (present/absent) three years after. Possible confounding factors at BL included: age, gender, disease duration (years), an education level (years), Montreal Cognitive Assessment (MoCA) (18), levodopa equivalent dose per day (LEDD) (19), and Unified Parkinson's Disease Rating Scale (UPDRS) subscale III (20). The confounding variables were not included in the regression analysis due to the small sample size.

### Neuropsychological and Neurological Evaluation

#### Anxiety Symptoms

Symptoms of anxiety were evaluated with the Beck Anxiety Inventory [BAI; German version; (21)]. BAI is a self-rating scale with 21 items. Items are evaluated on a four-point Likert scale ranging from 0 to 3 (e.g., not at all; a little; moderate; or many). The total score can range from 0 to 63 (higher scores represent increasing symptom severity). BAI has been validated by Leentjens et al. (22) for use in PD and a value > 13 has been identified to show clinically significant anxiety.

#### Depressive Symptoms

Depression symptoms were assessed by Beck Depressive Inventory-II [BDI-II; German version; (23)]. BDI-II is a valid and reliable tool in PD patients (24). It is a self-rating scale with 21 items, which are evaluated on a four-point Likert scale, ranging from 0 to 3 (e.g., “I do not feel sad”; “I feel sad much of the time”; “I am sad all the time”; “I am so sad and unhappy I can't stand it”). The total score can range from 0 to 63 (higher scores represent increasing symptom severity). Minimum range: 0–13; mild depression: 14–19, moderate depression: 20–28 and severe depression: 29–63. BDI-II has been validated for use in PD and a value > 14 has been identified to show clinically significant depression (24).

#### Apathy Symptoms

Symptoms of apathy were evaluated based on the Apathy Evaluation Scale (AES, German version; (25). This questionnaire is valid and reliable in PD patients (26). It consists of 18 items, which are evaluated on a four-point Likert scale, ranging from 0 to 3 with increasing severity (e.g., not at all; a little; moderate; and many). The total score can range from 0 to 54. More than 38 points indicate the presence of clinically relevant apathy symptoms.

#### UPDRS III

The severity of motor signs was assessed using the Unified Parkinson's Disease Rating Scale (UPDRS) (20) subscale III. This scale consists of 27 items that are rated on a five-level Likert scale ranging from 0 to 4 with increasing severity (normal; slight; mild; moderate and severe). The total score can range from 0 to 108. An increase in the score refers to severe difficulties.

#### Montreal Cognitive Assessment

The cognitive deficits were assessed using the Montreal Cognitive Assessment (MoCA) (18). MoCA is a valid and reliable tool for screening MCI in PD patients. The minimum to the maximum score of the 11 tasks carried out under the supervision of an expert ranges from 0 to 30; a score below 21 indicates dementia. MoCA is used in this study as a potential confounder and not as part of the formal outcome assessment of MCI.

#### Criteria for MCI

The neuropsychological assessment battery was used to classify MCI, based on the Movement Society Task Force Level II criteria according to Litvan et al. (5).

### Statistical Analyses

IBM SPSS Statistics for Windows, version 25.0 was used for the statistical analyses. Correlations were measured by eta-squared to assess the relationships between BL variables and MCI after three years of follow-up. The Eta coefficient test is a method for determining the correlation between a categorical variable and a scaled variable. The MCI data is a categorical variable with two levels: yes or no (MCI vs. non-MCI). Since the categorical variable data does not necessarily have a linear relationship with the scaled data, it is not possible to use Pearson's correlation coefficient (27). An Eta squared value ≥ of 0.14; ≥0.06 and ≥0.01 were considered a strong, moderate, and weak correlation, respectively. Spearman correlations were applied to assess the correlations between the independent variables.

Binary logistic regression analysis was performed to examine significant predictors of MCI. BAI, BDI-II, and AES were included in the model. Odds ratios (ORs) and 95% confidence intervals (CIs) were analyzed accordingly.

Data normality was verified using the Shapiro-Wilk test. The comparison of the medians between the two groups in BAI was carried out with the Mann-Whitney U tests. Besides, we generated ROC curves between BAI, AES, and BDI II tests as predictive factors for binary discrimination for NC patients who remained stable during the 3-year follow-up period and those who transitioned to MCI.

## Results

Twenty-nine participants completed the assessments (Table 1). Of the 29 patients, 11 patients (37.9%) had clinically significant anxiety levels with a cut-off score of at least 13. Based on the MCI Level II criteria, eight patients (27.58%; three female) developed MCI at the three-year follow-up.

**Table 1:** Participant's characteristics at Baseline.

**Sample (BL) with NC (*N* = 29)**	**Median [quartiles]^**a**^**
Age (years)	69 [63.5, 75]
Gender (female: male)	13:16
Education (years)	15 [12 17]
Disease duration (years)	6 [4, 9]
LEDD, mg/day	560 [299.75, 1026.63]
UPDRS III, points	18 [5, 25]
MoCA, points	27 [25, 29]
BAI	8 [4, 16]
BDI-II	7 [4, 11]
AES	25 [21, 30]

a *All values represent median [quartiles]. Quartiles refer to 25th and to the 75th percentile*.

*NC, normal cognition; BL, Baseline; LEDD, Levodopa-equivalent doses; UPDRS III, Unified Parkinson's Disease Rating Scale, Motor Subscale; MoCA, Montreal Cognitive Assessment; BAI, Beck Anxiety Inventory; BDI- II, Beck Depression Inventory-II; AES, Apathy Evaluation Scale*.

**Figure 1:**
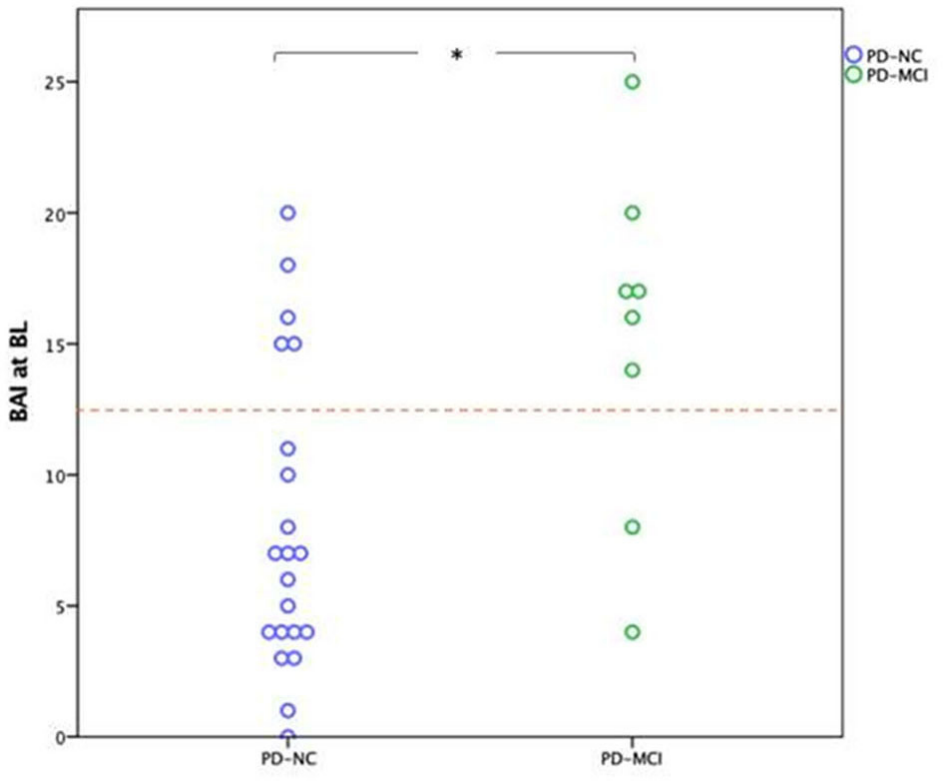
Dots represent the Baseline BAI scores of patients regarding their cognitive status at three years follow-up. The boxes include the median and the values between the 25th and 75th percentiles; *p < 0.01; The cutoff is 13. PD-NC, Parkinson's disease patients with normal cognitive; PD-MCI, Parkinson's disease patients with mild cognitive impairment; BAI, Beck Anxiety Inventory; BL, Baseline. AI scores of patients regarding their cognitive status at three years follow-up.

Patients who developed MCI three years later exhibited a median BAI of 16.5 with an interquartile range (9.5, 19.25) at BL. In the group who developed MCI after three years, 6 out of 8 patients had a BAI score of more than 13 on the BL. In the group with normal cognition after three years, 5 out of 21 patients had a BAI score of more than 13 on the BL (Figure 1).

Patients, with MCI three years later exhibited a median BDI-II of 9.5 with an interquartile range (7, 11.75) and a median AES of 29 with an interquartile range (21.5, 31.75) at BL.

Eta-squared, as well as Phi/Cramer-V showed that only BAI had a significant association with MCI status three years later (η^2^ = 0.23, *p* < 0.01). While BDI-II and AES showed no significant association (η^2^ = 0.06, *p* = 0.20) and AES (η^2^ = 0.02, *p* = 0.41). The same was true between MCI status and other baseline variables (Table 2).

**Table 2:** Correlations.

	**MCI**	**BAI**	**BDI-II**	**AES**	**MoCA**	**Age**	**Education**	**Dis. Duration**	**LEDD**	**Gender**	**UPDRS III**
MCI	1										
BAI	0.233^a^^**^	1									
BDI-II	0.061^a^	0.576^c^^**^	1								
AES	0.025^a^	−0.068^c^	−0.033^c^	1							
MoCA	0.091^a^	0.068^c^	−0.211^c^	−0.264^c^	1						
Age	0.000^a^	−0.133^c^	−0.018^c^	−0.047^c^	0.037^c^	1					
Education	0.049^a^	−0.159^c^	0.088^c^	−0.313^c^	−0.035^c^	0.495^c^^**^	1				
Disease Duration	0.000^a^	0.254^c^	0.100^c^	−0.067^c^	0.123^c^	−0.149^c^	−0.149^c^	1			
LEDD	0.017^a^	0.116^c^	−0.056^c^	0.127^c^	0.054^c^	−0.368^c^^*^	−0.164 ^c^	0.487^c^^**^	1		
Gender (male)	0.091^b^	0.354^c^	0.358^c^	0.062^c^	0.166^c^	−0.174^c^	−0.572^c^^**^	0.008^c^	−0.153^c^	1	
UPDRS_III	0.023^a^	0.341^c^	0.199^c^	−0.349^c^	0.194^c^	0.276^c^	0.305^c^	0.044^c^	0.054^c^	0.054^c^	1

*Correlations of MCI at three-year follow-up and the Baseline variables*.

** *p < 0.01*,

* *p < 0.05*.

a *Eta-squared*.

b *Phi/Cramer-V*.

c *Spearmen-Rho*.

*LEDD, Levodopa-equivalent doses; UPDRS III, Unified Parkinson's Disease Rating Scale, Motor Subscale; MoCA, Montreal Cognitive Assessment; BAI, Beck Anxiety Inventory; BDI-II, Beck Depression Inventory-II; AES, Apathy Evaluation Scale*.

Binary logistic regression analysis showed that a higher BL BAI score was an independent predictor for future development MCI (*OR* = 1.20, *p* = 0.02, *CI* = 1.03–1.41). No significant association was observed between BL BDI- II and MCI after three years (*OR* = 1.16, *p* = 0.19, *CI* = 0.95–1.46) and between BL AES and future development of MCI (*OR* = 1.06, *p* = 0.40, *CI* = 0.92–1.23).

**Figure 2:**
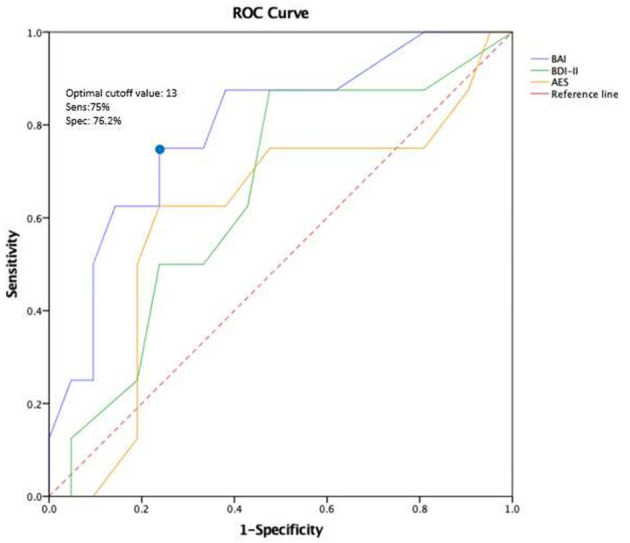
ROC curve of BAI, AES, and BDI-11 at baseline to predict MCI after three years of follow-up. Discriminant analysis based on the receiver operating characteristics (ROC) curve represents the predictive power of BAI, BDI-ll, and AES for the development of MCI. BAI, Beck Anxiety Inventory; BDI-11, Beck Depression Inventory-II; AES, Apathy Evaluation Scale.

Figure 2 shows the predictive power of BAI, BDI- II, and AES for the development of MCI, estimated by the area under the ROC curve. BAI significantly separated MCI status from cognitive normal status at three-year follow-up: The AUC under its ROC was 0.79 (*CI* = 0.61–0.98) and only it reached a significant level of *p* = 0.02; With a cut off score between 12.5 and 14.5 in the BAI, patients with MCI vs. non-MCI were separated with a sensitivity of 75.0% and a specificity of 76.2%. For our study, we chose a cut-off value of 13 points, which is also consistent with the standard cut-off value for the BAI.

To determine whether multicollinearity exists between the three neuropsychiatric symptoms (BAI, BDI-II, and AES), the tolerance statistics and variance inflation factor (VIF) statistics were analyzed. In this analysis, the lowest tolerance statistic was 0.65 and the highest VIF statistic was 1.53. Multicollinearity exists when the tolerance statistic is lower than 0.10 to 0.20 and the VIF statistic is > 5 to 10 (28). In our study, Multicollinearity was not found to be present.

## Discussion

The present study indicates, that MCI in PD is mainly predicted by levels of anxiety whereas neither depression nor apathy shows a significant association on future cognitive decline in our sample. Patients who developed MCI at three-year follow-up had higher scores of BAI at BL. In contrast, patients who did not develop MCI three years later initially presented lower BAI scores. These findings demonstrate an association between temporary primary anxiety and the subsequent development of MCI. This finding was also confirmed by ROC analyses: BL level of anxiety was the strongest risk factor for MCI and only this variable reached a significant level. This analysis showed that BAI with cut-off 13 used in our study allowed the detection of PD-MCI with a moderate to good sensitivity and specificity (22).

The fact that anxiety is strongly associated with cognitive impairment was detected in older persons with MCI (29), as well as in patients with PD (30). This association was specifically seen with the executive dysfunctions, specifically with impaired cognitive flexibility (29). This finding is consistent with the results of a former study (31). Numerous researchers have investigated the relationship between anxiety and cognition in PD patients (2, 30, 32).

Similarly, to the results in the present study, another study has shown that the initial level of anxiety in PD is predictive of cognitive decline after two years, mainly in the verbal and visual episodic memory (9). However, the primary aim of our study was not the association between anxiety, depression, apathy and cognitive impairment but their predictive value for the development of MCI. Contrary to former studies, we observed no predictive value of depression and apathy on future MCI. One possible explanation for this finding might be that, according to attentional control theory, anxiety leads to an increased focus on threatening stimuli, which disrupts the current task and negatively affects cognition (33). PD patients might be experiencing frequent threatening stimuli due to motor fluctuations between the “on” and “off” states, as anxiety increases when the dopaminergic medication wears off (34). Attention deficits may occur and over the long term reduce global cognitive function.

The link between anxiety and cognition may also be explained at the neurotransmitter level: Dopaminergic, noradrenergic, and serotonergic dysfunctions can be causes of both anxiety and cognition impairment in PD patients (3).

Our study has limitations as well as strengths. The strength of our study resides in its design. This study was designed as a longitudinal study to investigate the predictors of MCI in PD patients.

As a limitation, it should be mentioned that the study is based on a relatively small sample (*n* = 29 patients) and therefore has low power. In addition, because of the small sample size, we could not control for potential confounders (35). Another limitation may be that we used BAI rather than the Parkinson Anxiety Scale (PAS), a newer scale for measuring anxiety that better captures anxiety in PD patients (36). Therefore, we plan to use the PAS in future studies. In addition, different subtypes of MCI (amnestic and non-amnestic) could be analyzed to obtain a clearer understanding of the relationship between anxiety and MCI.

## Conclusions

In conclusion, our data support that anxiety is a potential risk factor for MCI in patients with PD.

## Data Availability Statement

The raw data supporting the conclusions of this article will be made available by the authors, without undue reservation.

## Ethics Statement

The studies involving human participants were reviewed and approved by Ethical Committee Basel. The patients/participants provided their written informed consent to participate in this study.

## Author Contributions

KT: study conception and design, data analyses, interpretation, and drafting of the manuscript. AM: data analyses, interpretation, drafting of the manuscript, and review and critical revision of the manuscript for important intellectual content. SB and PF: review and critical revision of the manuscript for important intellectual content. RL: drafting of the manuscript and review and critical revision of the manuscript for important intellectual content. UG: study conception and design, drafting of the manuscript, and review and critical revision of the manuscript for important intellectual content. All authors contributed to the article and approved the submitted version.

## Conflict of Interest

The authors declare that the research was conducted in the absence of any commercial or financial relationships that could be construed as a potential conflict of interest.

## Publisher's Note

All claims expressed in this article are solely those of the authors and do not necessarily represent those of their affiliated organizations, or those of the publisher, the editors and the reviewers. Any product that may be evaluated in this article, or claim that may be made by its manufacturer, is not guaranteed or endorsed by the publisher.
