# Supplementary material for: Anxiety, Depression, and Apathy as Predictors of Cognitive Decline in Patients With Parkinson's Disease—A Three-Year Follow-Up Study
Source: Front Neurol. 2022 Feb 8;13:792830. doi: 10.3389/fneur.2022.792830 (PMC8860828; doi:10.3389/fneur.2022.792830)
Supplement: Supplementary file 1 [file Data_Sheet_1.docx]

**Supplementary material**

In the present study, Mild Cognitive Impairment (PD-MCI) was evaluated along the Movement Disorder Society Task Force guidelines for the diagnosis of PD-MCI [1]; in our assessment, we applied Level II diagnostic criteria. Neuropsychological impairment was rated as positive if a patient scored below 1.5 standard deviations when compared to the normative data in at least two cognitive tests; represented by either two impaired tests in one out of five cognitive domains or one impaired test in two different cognitive domains. Additionally, for the MCI diagnosis a significant decline in cognition from premorbid level is required. In the present study, significant cognitive decline was rated as positive if either an informant indicated an IQCODE score >3.29 [2] and/or the patient reported cognitive changes in an interview.

The definition of neuropsychological impairment was based on performance in five domains in the following cognitive tests:

**Attention and Working Memory**

Stroop test, naming colors [3]

Trail Making Test, part A [4]

Digit Span, backwards [5]

**Executive functions**

Trail Making Test, ratio (part B/A: [4])

Wisconsin Card Sorting test, number of errors [6]

Phonemic fluency, number of correct words [7]

**Language**

Boston Naming Test, number of correct words [8]

Similarities, correct answers [5]

**Memory**

California Verbal learning Test, Long-delay free recall [9]

California Verbal learning Test, Recognition-discriminability [9]

**Visuo-spatial**

Block Design Test [5]

Rey-Osterrieth Complex Figure, Copy [10]

**References**

[1] I. Litvan, J.G. Goldman, A.I. Tröster, B.A. Schmand, D. Weintraub, R.C. Petersen, B. Mollenhauer, C.H. Adler, K. Marder, C.H. Williams‐Gray, D. Aarsland, J. Kulisevsky, M.C. Rodriguez‐Oroz, D.J. Burn, R.A. Barker, M. Emre, Diagnostic criteria for mild cognitive impairment in Parkinson’s disease: Movement Disorder Society Task Force guidelines, Movement Disorders. 27 (2012) 349–56. https://doi.org/10.1002/mds.24893.

[2] M.M. Ehrensperger, M. Berres, K.I. Taylor, A.U. Monsch, Screening properties of the German IQCODE with a two-year time frame in MCI and early Alzheimer’s disease, Int Psychogeriatr. 22 (2010) 91–100. https://doi.org/10.1017/S1041610209990962.

[3] Stroop J R, Studies of interference in serial verbal reactions, Journal of Experimental Psychology. 18 (1935) 643–662.

[4] R.M. Reitan, VALIDITY OF THE TRAIL MAKING TEST AS AN INDICATOR OF ORGANIC BRAIN DAMAGE, Perceptual and Motor Skills. 8 (1958) 271–276. https://doi.org/10.2466/pms.1958.8.3.271.

[5] C. Härtig, H.J. Markowitsch, H. Neufeld, P. Calabrese, K. Deisinger, J. Kessler, Wechsler Gedächtnis Test - Revidierte Fassung, Verlag Hans Huber, Bern, 2000.

[6] H.E. Nelson, A modified card sorting test sensitive to frontal lobe defects, Cortex. 12 (1976) 313–324.

[7] L.L. Thurstone, Primary mental abilities, Science. 108 (1948) 585.

[8] J.C. Morris, A. Heyman, R.C. Mohs, J.P. Hughes, G. van Belle, G. Fillenbaum, E.D. Mellits, C. Clark, The Consortium to Establish a Registry for Alzheimer’s Disease (CERAD). Part I. Clinical and neuropsychological assessment of Alzheimer’s disease, Neurology. 39 (1989) 1159–1165.

[9] D. Delis, J. Kramer, B. Ober, E. Kaplan, The California Verbal Learning Test: Administration and interpretation, in: Psychological Corporation, San Antonio, TX, 1987.

[10] J.F. Duley, J.W. Wilkins, S.L. Hamby, D.G. Hopkins, R.D. Burwell, N.S. Barry, Explicit scoring criteria for the Rey-Osterrieth and Taylor complex figures, Clinical Neuropsychologist. 7 (1993) 29–38. https://doi.org/10.1080/13854049308401885.
